# Convergent evolution of heat-inducibility during subfunctionalization of the Hsp70 gene family

**DOI:** 10.1186/1471-2148-13-49

**Published:** 2013-02-21

**Authors:** Sascha Krenek, Martin Schlegel, Thomas U Berendonk

**Affiliations:** 1 Institute of Hydrobiology, Technische Universität Dresden, Dresden, 01062, Germany; 2 Molecular Evolution and Animal Systematics, University of Leipzig, Leipzig, 04103, Germany

**Keywords:** Ciliate, Convergent evolution, DnaK, Gene duplication, Heat-inducibility, Heat-shock proteins, Molecular chaperones, *Paramecium*, RT-qPCR, Temperature stress

## Abstract

**Background:**

Heat-shock proteins of the 70 kDa family (Hsp70s) are essential chaperones required for key cellular functions. In eukaryotes, four subfamilies can be distinguished according to their function and localisation in different cellular compartments: cytosol, endoplasmic reticulum, mitochondria and chloroplasts. Generally, multiple cytosol-type Hsp70s can be found in metazoans that show either constitutive expression and/or stress-inducibility, arguing for the evolution of different tasks and functions. Information about the *hsp70* copy number and diversity in microbial eukaryotes is, however, scarce, and detailed knowledge about the differential gene expression in most protists is lacking. Therefore, we have characterised the Hsp70 gene family of *Paramecium caudatum* to gain insight into the evolution and differential heat stress response of the distinct family members in protists and to investigate the diversification of eukaryotic *hsp70*s focusing on the evolution of heat-inducibility.

**Results:**

Eleven putative *hsp70* genes could be detected in *P. caudatum* comprising homologs of three major Hsp70-subfamilies. Phylogenetic analyses revealed five evolutionarily distinct Hsp70-groups, each with a closer relationship to orthologous sequences of *Paramecium tetraurelia* than to another *P. caudatum* Hsp70-group. These highly diverse, paralogous groups resulted from duplications preceding *Paramecium* speciation, underwent divergent evolution and were subject to purifying selection. Heat-shock treatments were performed to test for differential expression patterns among the five Hsp70-groups as well as for a functional conservation within *Paramecium.* These treatments induced exceptionally high mRNA up-regulations in one cytosolic group with a low basal expression, indicative for the major heat inducible *hsp70*s. All other groups showed comparatively high basal expression levels and moderate heat-inducibility, signifying constitutively expressed genes. Comparative EST analyses for *P. tetraurelia hsp70*s unveiled a corresponding expression pattern, which supports a functionally conserved evolution of the Hsp70 gene family in *Paramecium*.

**Conclusions:**

Our analyses suggest an independent evolution of the heat-inducible cytosol-type *hsp70*s in *Paramecium* and in its close relative *Tetrahymena*, as well as within higher eukaryotes. This result indicates convergent evolution during *hsp70* subfunctionalization and implies that heat-inducibility evolved several times during the course of eukaryotic evolution.

## Background

The environmental stress response in all organisms as diverse as pro- and eukaryotes is generally coupled with a remarkable change in gene expression patterns and an enhanced synthesis of several ‘stress proteins’ [1]. Because they were first described in *Drosophila melanogaster* larvae that were accidentally exposed to elevated temperatures [2], these stress-related proteins were called heat-shock proteins (Hsps). Extensive research on Hsps revealed also a constitutive expression of some members of these proteins, suggesting that they are also essential in maintaining the cellular functions under normal physiological conditions. These members are therefore designated as heat-shock cognate proteins (Hscs) [3,4]. Furthermore, Hsps respond not only to increased temperatures, but also chemicals, heavy metals, UV light, hypoxia and other stressors can induce their synthesis [5,6].

Some of the most important and well investigated Hsps are the members of the 70 kDa heat-shock protein family (Hsp70s). They belong to the highest conserved proteins and are present in almost all species, except for some archaea. Prokaryotic Hsp70 (DnaK) proteins share about 50% amino acid identity with eukaryotic Hsp70s. All known Hsp70 proteins exhibit highly conserved amino acid sequences and domain structures, such as a conserved N-terminal ATPase domain, a region with protease sensitive sites and a peptide binding domain at the C-terminal region (reviewed in e.g. [7-9]). Due to this high conservation, Hsp70 has been widely used as a suitable phylogenetic marker in molecular evolution. It has been applied to deep phylogenetic relationships (such as between animals and fungi) or to confirm the monophyly of Metazoa [10], as well as relationships between archaea and Gram-positive bacteria or Gram-negative bacteria and eukaryotes [11-13]. Hsp70 genes and proteins have also been used for phylogenetic studies of different protozoan parasites such as *Trypanosoma* or *Leishmania*[14,15], as well as of non-parasitic protozoans such as *Euplotes* or *Paramecium*[16,17].

In eukaryotes, several of these Hsp70 proteins that belong to four subfamilies are encoded by the nuclear genome (reviewed in e.g. [18,19]). The discrimination of the four Hsp70 subfamilies corresponds to the intracellular localisation of the Hsp70 proteins in the major compartments of the cell: the cytosol, the endoplasmic reticulum (ER) and the organelles mitochondria and chloroplasts [11]. These multigene family members emerged from numerous duplication events or replicative transpositions and evolved by a combination of birth-and-death processes, gene conversion events and purifying selection (e.g. [20-22]). Therefore, caution is called for when using Hsp70s as phylogenetic marker, because paralogy can distort phylogenetic relationships [23]. While the evolution of all Hsp70 family members is not yet completely understood, the pathways for the evolution of the nucleus-encoded Hsp70s of the cell organelles are well known. The mitochondrial and the chloroplast homologs are derivatives from an endosymbiotic alphaproteobacterium and cyanobacterium, respectively, followed by a subsequent horizontal gene transfer of the *hsp70* homologs to the nucleus. Further, the evolutionary relationship between the ER- and cytosol-type *hsp70*s indicates that these genes have arisen very early in the common eukaryotic ancestor by gene duplication [11,12,24].

All members of the Hsp70 family carry out molecular chaperone functions that facilitate correct protein folding, as well as membrane translocation and the subsequent refolding of proteins [25]. Through the course of evolution, these multiple Hsp70 family members, which can be found in pro- and eukaryotes, have acquired different chaperone tasks and functions (e.g. [26,27]). For example, in all multicellular eukaryotes studied so far, some cytosol-type members show a constitutive expression under normal physiological conditions (Hsc70s) while others are highly inducible (Hsp70s) in consequence of several stress conditions (below, we will use the term Hsp70 to refer to both the inducible and constitutive 70 kDa chaperones). Detailed knowledge about the number, diversity and differential gene expression of the Hsp70 family members in most unicellular eukaryotes is, however, scarce. For example, the yeast genome of *Saccharomyces cerevisiae* encodes fourteen Hsp70-like genes [28] comprising cytosol-type *hsp70*s that show constitutive expression (SSA1, SSA2) as well as heat-inducibility (SSA1, SSA3; e.g. [3,29]). Further, the genome sequencing projects of the ciliate species and model eukaryotes *Tetrahymena thermophila*[30] and *Paramecium tetraurelia*[31] unveiled several putative *hsp70* genes with conserved Hsp70 domains. In *Paramecium*, whole genome duplications and subsequent gene loss events have shaped the Hsp70 gene family [31], but it is still uncertain whether subfunctionalization or gene dosage constraints are the main forces for *hsp70* duplicate retention. While the major constitutively expressed and heat-inducible cytosol-type *hsp70*s have been identified for *T. thermophila*[32], the differential expression pattern of the Hsp70 family in *P. tetraurelia* has remained unclear. Additionally, the evolutionary relationships of the *hsp70* homologs from these closely related microbial eukaryotes are vague. Therefore, we have investigated the Hsp70 multigene family of another paramecia species, *Paramecium caudatum*, by sequencing an *hsp70* cDNA clone library.

Based on the resultant sequence data, we performed phylogenetic and comparative sequence analyses to gain insight into the evolution of the Hsp70 gene family in *Paramecium.* To disentangle the evolutionary relationships among ciliate *hsp70*s, which are in certain cases affected by whole genome duplications, we included not only a comprehensive set of homologous *hsp70*s of different pro- and eukaryotes, but also data from recent ciliate genome projects: *Tetrahymena*, *Ichthyophthirius* and *Oxytricha*. We further developed a target specific RT-qPCR assay for *P. caudatum hsp70*s in order to demonstrate an expected differential gene expression among the family members and to distinguish constitutively expressed and heat-induced *hsp70*s. Using these data, we tested for a hypothesised functional conservation of *Paramecium hsp70* orthologs via comparative EST analyses and investigated the diversification of eukaryotic *hsp70*s to clarify the evolution of heat-inducibility within the 70 kDa molecular chaperone family.

## Results

### Characterisation of *P. caudatum hsp70*s

Sequencing of sixty putative *hsp70* gene fragments, amplified from cDNA with degenerated primers, revealed eleven different *hsp70* nucleotide sequences (1363bp – 1378bp) from the Hsp70/DnaK-family of *Paramecium caudatum.* When the majority of the obtained sequences became redundant, we stopped sequencing of further clones of our *hsp70* cDNA clone library. Therefore, we cannot exclude the possibility of additional homologs expressed in *P. caudatum*, but a sequence homology search (BLASTn/p) clearly assigned the eleven homologs to the three major Hsp70-subfamilies: cytosol (CY), endoplasmic reticulum (ER) and mitochondria (MT). These analyses further revealed the expression of one MT, five CY and five ER related Hsp70 proteins in *P. caudatum*. The sequences were designated according to their origin and sequence similarity as follows: *PcHsp70CY1a*, *PcHsp70CY1b*, *PcHsp70CY1c*, *PcHsp70CY2a*, *PcHsp70CY2b*, *PcHsp70ER1a*, *PcHsp70ER1b*, *PcHsp70ER2a*, *PcHsp70ER2b*, *PcHsp70ER2c*, *PcHsp70MT1a*.

The homologs *PcHsp70CY1a* and *PcHsp70CY1b* differed at only 4 nucleotide positions, and encode the same amino acid sequence. The homologs *PcHsp70ER1a* and *PcHsp70ER1b* differed by only one ‘C’ to ‘T’ substitution, resulting also in the same amino acid sequence. *PcHsp70ER2a*, *PcHsp70ER2b* and *PcHsp70ER2c* showed 1 to 3 nucleotide substitutions, but were indiscriminative at the amino acid level too (Additional file 1: Table S1). Because we used a proofreading polymerase for PCR amplification and a high fidelity RNA-dependent DNA polymerase for cDNA synthesis, these nucleotide substitutions can be considered authentic. The analyses of the deduced amino acid sequences revealed seven different homologous sequences, with an averaged pairwise distance of 0.38 and a maximum pairwise distance of 0.66. The amplified fragments for all CY homologs as well as *PcHsp70ER1a* and *PcHsp70ER1b* encode 459 amino acids (aa); *PcHsp70ER2a*, *PcHsp70ER2b* and *PcHsp70ER2c* encode 457 aa and *PcHsp70MT1a* encodes 454 aa.

**Figure 1:**
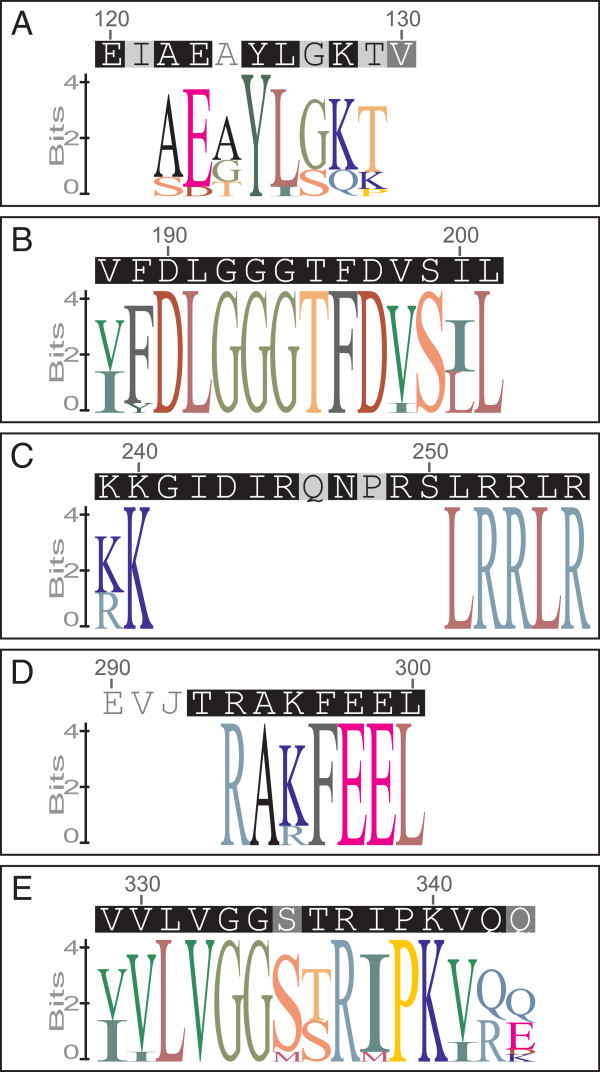
**Amino acid sequence logos of *P. caudatum *Hsp70 motifs.** (**A**) putative ATP/GTP-binding site motif A (P-loop), (**B**) Hsp70 family signature 2, (**C**) putative bipartite nuclear localization signal, (**D**) potential signature for eukaryotic non-organellar Hsp70 proteins, and (**E**) Hsp70 family signature 3. The size of each letter is proportional to the frequency of occurrence of the respective amino acid in a multiple sequence alignment. The letters are sorted with the most frequent one on top and the overall height of each stack indicates the information content of the sequence at the given position (0–4 bits). The sequence logos were prepared from sequences of all *P. caudatum hsp70*s (**A**,**B**,**E**), from the cytosolic and ER-type homologs (**D**), and from the cytosolic *hsp70* genes only (**C**).

All identified sequences shared two amino acid signature motifs (Figure 1B/E; see also Additional file 2: Figure S1), which are highly conserved regions and present in all eukaryotic Hsp70 family members (note: the forward primer Hsp70ForDeg targeted the Hsp70 family signature 1). The consensus sequences are as follows: Hsp70 family signature 2 [VI]-[FY]-D-L-G(3)-T-F-D-[VI]-S-[IL]-L and Hsp70 family signature 3 [VI]-[VI]-L-V-G(2)-[SM]-[TS]-R-[IM]-P-K-[VI]-[QR]-[QEDK], where the square brackets show a list of acceptable amino acids at the given position and the numerical values between parentheses indicate repetition of the respective amino acid [33]. Another conserved signature, the putative ATP/GTP-binding site motif A (P-loop), could be identified in all eleven homologs, albeit with relatively high variation (Figure 1A, see also Additional file 2: Figure S1). As expected, the putative bipartite nuclear localization signal, a targeting sequence essential for the selective translocation of nucleo-/cytoplasmatic proteins into the nucleus, could be detected for the CY homologs only [34]. Its consensus sequence is [KR]-K-x(10)-L-R(2)-L-R, where the symbol ‘x’ indicates a position of any acceptable amino acid (Figure 1C, see also Additional file 2: Figure S1). Additionally, all CY and ER homologs possess the R-A-[KR]-F-E-E-L consensus motif that serves as a potential signature for eukaryotic non-organellar Hsp70 proteins [35].

### Evolutionary relationships

**Figure 2:**
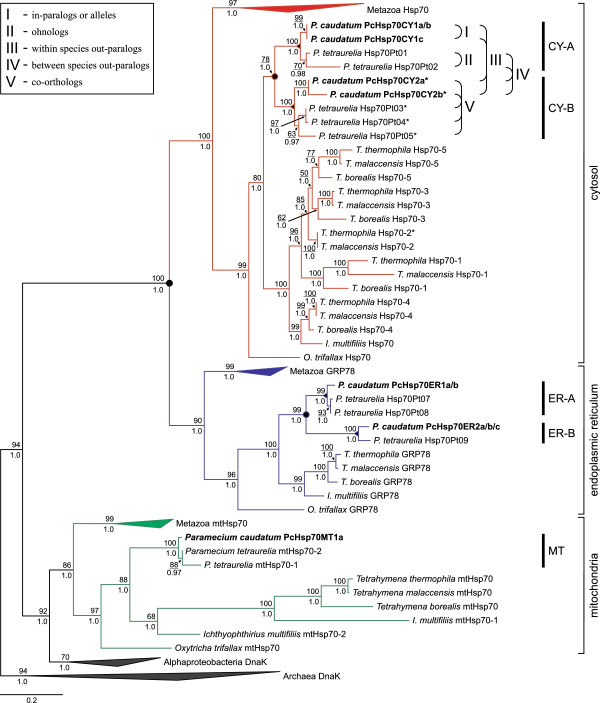
**Phylogenetic reconstruction of homologous *hsp70 *sequences from different pro- and eukaryotes.** The alignment was based on a comprehensive amino acid alignment containing 97 homologs that show the typical Hsp70 family signatures. Sequences obtained within this study are shown in boldface. The alignment length was constricted to 489 amino acids, including gaps. The illustrated tree is based on the Maximum-Likelihood calculations using the LG+I+*Γ* protein evolution model with 1,000 rapid bootstrap replicates. The Archaea DnaK sequences served as outgroup. Numbers at the nodes (occasionally indicated by an arrow) represent support values for the Maximum-Likelihood (above line) and Bayesian analysis (below line), respectively. The three major Hsp70-subfamilies—cytosol (CY), endoplasmic reticulum (ER) and mitochondria (MT)—are indicated by squared brackets to the right. Vertical bars to the right of the tree designate the five different Hsp70-groups (CY-A, CY-B, ER-A, ER-B, MT) detected in *Paramecium*. Highly heat inducible cytosol-type *hsp70* genes (if known) are specified by an asterisk. Filled circles at the nodes represent ancient and intermediary gene duplication events, while filled triangles at the nodes point to speciation within *Paramecium*. Rounded brackets to the right exemplify evolutionary relationships among the *Paramecium* Hsp70 homologs and are defined in the upper left box frame. See Additional file 4: Figure S2 for the uncollapsed tree and its electronic version deposited in TreeBASE under accession number TB2:S13746.

As illustrated in the phylogenetic Hsp70 tree in Figure 2 showing the three major Hsp70-subfamilies (CY, ER, MT) among different eukaryotes, the ascertained *P. caudatum* sequences integrated well within the separate subfamilies with high support values. In addition, all ciliate sequences clustered into clades in which the *Paramecium* homologs formed monophyla. These analyses also revealed the existence of different Hsp70-groups within the different *P. caudatum* Hsp70 subfamilies. Here, two distinct Hsp70-groups within the cytosolic as well as the ER-type subfamily could be detected. Each of these *P. caudatum* Hsp70-groups from the same subfamily (CY or ER) showed a closer relationship to orthologous Hsp70 sequences of *Paramecium tetraurelia* than to another *P. caudatum* Hsp70-group. The group CY-A consisted of the three in-paralogs *PcHsp70CY1a*, *PcHsp70CY1b* and *PcHsp70CY1c*; the group CY-B contained *PcHsp70CY2a* and *PcHsp70CY2b*; the group ER-A comprised the genes *PcHsp70ER1a* and *PcHsp70ER1b*; and group ER-B included *PcHsp70ER2a*, *PcHsp70ER2b* and *PcHsp70ER2c*. The mitochondrial homolog *PcHsp70MT1a* was determined as group MT for further analyses, even though we could detect only one mitochondrial *hsp70* gene sequence. Since we cannot confirm complete locus homozygosity of the clonal *P. caudatum* strain that we used in this study, the detected within-group paralogs (in-paralogs) might constitute alleles. However, the amitotic division of the ciliate macronucleus (MAC) during vegetative growth can lead to an unequal partitioning of the alleles to daughter MACs [36], resulting in the expression of only one of the two alleles. In conjunction with the detected low mutation rate in *Paramecium*[37] and the comparatively high sequence divergence of the CY-B in-paralogs (2.5%), we would argue in this case for in-paralogs rather than alleles.

The detection of eleven putative *hsp70* homologs in *P. caudatum* is in concordance with former studies on the Hsp70 multigene family in other ciliate species (cf. [16]). The *P. tetraurelia* genome project [31,38], for example, revealed fifteen putative *hsp70* sequences in MAC DNA, while only thirteen possess the typical conserved domain structures with an N-terminal ATPase and a C-terminal peptide-binding domain. However, our motif analyses revealed that only ten homologs exhibit all three Hsp70 family signatures, indicating the existence of potential *hsp70* pseudogenes in the *P. tetraurelia* MAC genome. In addition, genome analyses for *Tetrahymena thermophila* revealed thirteen putative *hsp70* genes featuring conserved Hsp70 domains [32]. Similar to *P. tetraurelia*, only seven hsp70 homologs comprise the three Hsp70 family signatures. Interestingly, while we could find all orthologous sequences of these seven *T. thermophila hsp70*s in its close relative *T. malaccensis* by utilizing the *Tetrahymena* Comparative Database, we could not detect the heat-inducible ortholog of *hsp70-2* in *T. borealis* (see Figure 2). Furthermore, in *Ichthyophthirius multifiliis* and *Oxytricha trifallax* we could detect only one CY- and one ER-type *hsp70* homolog comprising all three Hsp70 family signatures.

### Modes of evolution

The five putative Hsp70-groups detected in *Paramecium* showed consistent phylogenetic patterns according to the assumed evolutionary relationship between the species (Figure 2), thereby supporting the model of divergent evolution in the *Paramecium* Hsp70 gene family. Additionally, a codon-based Z-test [39] on all *P. caudatum hsp70*s revealed evidence for purifying selection, which is indicated by higher numbers of synonymous (*d*_*S*_) to non-synonymous (*d*_*N*_) nucleotide differences per site in pairwise comparisons (Table 1). While the paralogs between the two Hsp70-groups per subfamily (‘within species out-paralogs’, cf. Figure 2) show a comparatively high amino acid divergence of up to 19%, the potential paralogs within each group (‘in-paralogs’, cf. Figure 2) show a rather low nucleotide diversity (0.1 – 2.5%). This high similarity of the in-paralogs could be indicative of very recent duplication events or might be explained by purifying selection (*d*_*N*_ <*d*_*S*_) or gene conversion (*d*_*N*_ = *d*_*S*_). The results of a codon-based Z-test for neutral evolution revealed that the null hypothesis of strict-neutrality (*d*_*N*_ = *d*_*S*_) could not be rejected for the CY-B and ER-type in-paralogs (Additional file 3: Table S2), while the sequence similarity among the CY-A in-paralogs seems to be caused by purifying selection (*d*_*N*_ <*d*_*S*_). A test for gene conversion events revealed significant evidence (*p* < 0.05) for only two very short regions (18bp and 53bp) between out-paralogs, which might in fact be misidentifications due to possible misalignment artefacts. Please note that the substitutions within the ER and CY-A groups are very few and therefore do not allow for definite conclusions of the within-group comparisons; on the other hand, the discrepancy between the CY-A and CY-B group is obvious and implies different modes of evolution.

**Table 1:** **Estimates of codon-based evolutionary divergence between *P. caudatum *Hsp70 genes**

	***Paramecium caudatum *PcHsp70**										
	**CY1a**	**CY1b**	**CY1c**	**CY2a**	**CY2b**	**ER1a**	**ER1b**	**ER2a**	**ER2b**	**ER2c**	**MT1a**
PcHsp70											
CY1a		0.00	0.00	0.10	0.12	0.27	0.27	0.29	0.29	0.29	0.31
CY1b	0.01		0.00	0.10	0.12	0.27	0.27	0.29	0.29	0.29	0.31
CY1c	0.03	0.02		0.10	0.12	0.27	0.27	0.29	0.29	0.29	0.31
CY2a	0.76	0.75	0.74		0.02	0.26	0.26	0.28	0.28	0.28	0.30
CY2b	0.77	0.76	0.75	0.03		0.26	0.26	0.28	0.28	0.28	0.31
ER1a	0.65	0.65	0.65	0.62	0.61		0.00	0.13	0.13	0.13	0.34
ER1b	0.65	0.65	0.65	0.62	0.61	0.00		0.13	0.13	0.13	0.34
ER2a	0.66	0.66	0.65	0.61	0.64	0.57	0.58		0.00	0.00	0.35
ER2b	0.66	0.66	0.65	0.61	0.64	0.57	0.57	0.01		0.00	0.35
ER2c	0.65	0.65	0.65	0.61	0.64	0.57	0.57	0.01	0.00		0.35
MT1a	0.65	0.64	0.65	0.58	0.59	0.62	0.62	0.64	0.64	0.64	

The number of synonymous differences per synonymous site (*d*_*S*_) (below diagonal) and of nonsynonymous differences per nonsynonymous site (*d*_*N*_) (above diagonal) for comparisons of eleven *P. caudatum hsp70* homologs are shown. Analyses were conducted using the Nei-Gojobori model included in MEGA5. All ambiguous positions were removed for each sequence pair with a total of 465 amino acid positions in the final dataset.

### Differential gene expression

The group classification with CY-A, CY-B, ER-A, ER-B and MT was used for the expression analyses, since RT-qPCR primers and MGB™-probes were specifically designed to amplify or bind the respective *hsp70* sequences within one group. The stability analysis of the reference genes *GAPDH* and *EF-1α* using the geNorm v.3.5 applet [40] revealed a high stability of both genes. The averaged expression stability value was 0.054 and the pairwise variation value (0.115) was below the proposed cut-off value of 0.15. We therefore used these two genes to normalise the *hsp70* mRNA levels in all further analyses.

**Figure 3:**
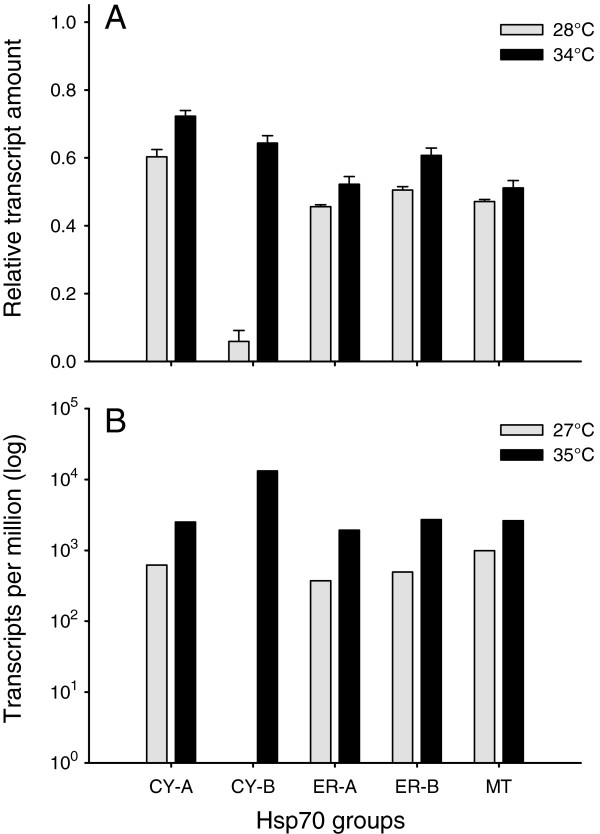
**Relative expression amounts of different Hsp70-groups in *Paramecium*.** (**A**) Relative transcript quantity of the *P. caudatum* Hsp70-groups CY-A, CY-B, ER-A, ER-B and MT at optimum temperature (28°C) and after heat shock (34°C) were assessed by RT-qPCR; levels were normalised to the highest and lowest observed cycle threshold (Ct) value of the whole data set and expressed in arbitrary units as relative mRNA amount (lowest Ct value = 1.0, highest Ct value = 0.0). All values are shown as mean ± standard error (n = 5). (**B**) Relative transcript amount of Hsp70-groups derived from EST libraries of *P. tetraurelia* cultured at 27°C or 35°C. Library screening was performed using the Local Blast engine in BioEdit (e-value cut-off of e < E^-100^) for orthologous EST sequences of the five *P. caudatum* Hsp70-groups using reference nucleotide sequences of *P. tetraurelia*. Resulting EST counts were used to calculate relative expression levels of the five Hsp70-groups in transcripts per million.

As illustrated in Figure 3A, the CY-A relative expression level (*rel*_CY-A_ = 0.60) at optimum temperature (28°C) was the highest among all five Hsp70-groups. While the basal expression levels of the ER-A, ER-B and MT group were comparatively similar (*rel*_ER-A_ = 0.46, *rel*_ER-B_ = 0.51, *rel*_MT_ = 0.47), the CY-B levels instead were considerably lower (*rel*_CY-B_ = 0.06), indicating that these genes (*PcHsp70CY2a* and *PcHsp70CY2b*) were transcribed at low basal levels under normal physiological conditions. After heat-shock exposure for two hours at 34°C, we could detect changes of the *hsp70* transcript levels (Figure 3A). While the reference genes *GAPDH* and *EF-1α* showed comparatively stable expression levels between 28°C (*rel*_GAPDH_ = 0.70, *rel*_EF-1α_ = 0.98) and 34°C (*rel*_GAPDH_ = 0.67, *rel*_EF-1α_ = 0.95), the heat shock provoked an induction of all different Hsp70-groups. After heat shock, the relative transcription level of CY-A (*rel*_CY-A_ = 0.72) was higher than that of *GAPDH* (*rel*_*GAPDH*_ = 0.67) and still showing the highest *hsp70* mRNA expression level. The CY-B group, however, was highly induced (*rel*_CY-B_ = 0.64), resulting in the second most expressed Hsp70-group.

**Figure 4:**
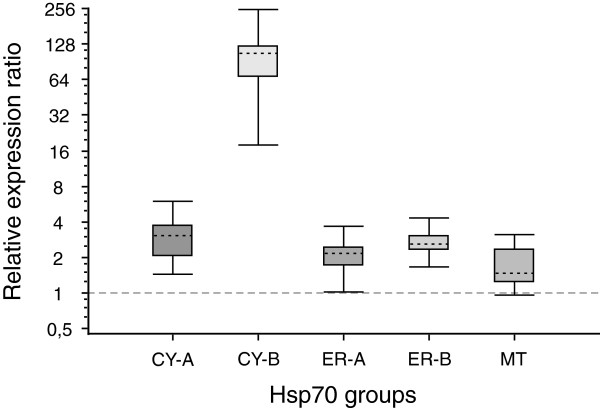
**Relative mRNA transcript induction of the five *P. caudatum *Hsp70-groups after heat shock.** Expression ratios were assessed by RT-qPCR using 28°C as control treatment and two-hour incubation at 34°C as heat-shock treatment. Transcript levels of the Hsp70-groups CY-A, CY-B, ER-A, ER-B and MT were normalised to the reference genes *GAPDH* and *EF-1a.* Relative expression ratios (fold-change) were calculated using the PCR-efficiency based method in the programme REST 2009. Results are illustrated as Whisker-box plots where the dotted line represents the sample median (n = 5) and the box area encompasses 50% of all observations. The grey dashed line defines the value of no change in relative expression.

Performing relative expression analyses using REST 2009 unveiled a significant up-regulation of all Hsp70-groups (Figure 4). While the transcription level of the CY-A, ER-A, ER-B and the MT group were slightly up-regulated between 1.7- and 3.0-fold, CY-B was strikingly induced by thermal stress with an averaged up-regulation of 84.2-fold. This indicates that the CY-B genes (*PcHsp70CY2a* and *PcHsp70CY2b*) are the major inducible forms of the Hsp70 multigene family in *P. caudatum*. Note that the fairly high standard errors as indicated in the Whisker-Box plots in Figure 4 resulted from the inclusion of the PCR efficiency into the relative expression calculation of REST 2009.

The screening of two *P. tetraurelia* EST libraries for orthologous sequences of the detected five *P. caudatum* Hsp70-groups revealed that all corresponding *P. tetraurelia hsp70*s seem to be up-regulated during heat exposure to 35°C, compared to normal physiological conditions at 27°C (Figure 3B). While EST counts for CY-B related genes could not be detected at 27°C, these genes were highly abundant at 35°C. Therefore, the CY-B related homologs seem to represent the major heat inducible *hsp70* genes of this multigene family in *P. tetraurelia* as well.

## Discussion

Phylogenetic analyses based on an Hsp70 amino acid alignment reliably assigned the detected *P. caudatum hsp70* genes to the respective Hsp70-subfamilies cytosol (CY), endoplasmic reticulum (ER) and mitochondria (MT) (Figure 2). These analyses further showed not only that these homologs can be divided into the three subfamilies, but they also revealed the existence of five putative Hsp70-groups in *P. caudatum*: one MT group consisting of only one homolog, but two distinct groups within the CY-, as well as two groups within the ER-type subfamily. Here, each CY- and ER-type Hsp70-group shows a closer relationship to putative orthologous sequences of *P. tetraurelia* than to the other *P. caudatum* Hsp70-group of the same subfamily (Figure 2). These findings suggest a gene duplication event before the speciation of *P. caudatum* and *P. tetraurelia,* but obviously after the divergence of *Paramecium* and *Tetrahymena* (another closely related oligohymenophorean ciliate) since both *Paramecium* CY- and ER-type homologs form monophyletic clades (see Figure 2). Therefore, the last common ancestor of *Paramecium* and *Tetrahymena* should have had one functional CY- and one ER-type *hsp70* homolog, while the common ancestor of *P. caudatum* and *P. tetraurelia* has possessed one functional gene of each of the four non-organellar Hsp70-groups, meaning one CY-A, CY-B, ER-A and ER-B homolog.

Using an RT-qPCR approach, we could show considerable differences among the five assigned Hsp70-groups of *Paramecium caudatum*. These analyses revealed the group CY-A as the major constitutively expressed Hsp70-group in *P. caudatum* at optimum physiological temperatures. Even though this study showed a significant up-regulation in mRNA levels of the CY-A group members after heat shock (~3.0-fold), this does not necessarily cause the rejection of their Hsc70 (heat-shock cognate protein 70) affiliation, since many constitutively expressed *hsp70*s can be induced under specific conditions (e.g. [41-44]). We have also seen that the Hsp70-group CY-B holds a very low mRNA abundance at optimum growth temperatures, but was highly induced after heat shock (~84-fold). This induction resulted in the second most abundant Hsp70s during heat stress and suggests that the two genes *PcHsp70Cy2a* and *PcHsp70Cy2b* of group CY-B represent the major heat-inducible homologs in *P. caudatum*.

As previously mentioned, the *P. caudatum* Hsp70-groups CY-A and CY-B showed a closer relationship to orthologous Hsp70 sequences of *P. tetraurelia* than to each other. The comparative analysis of two *P. tetraurelia* EST libraries (constructed from RNA of cells grown at 27°C or 35°C) revealed comparable expression patterns between the *P. caudatum* and the *P. tetraurelia* Hsp70-groups (cf. Figure 3A and 3B). Here, the CY-A group homologs are also highly expressed under normal physiological conditions, indicating constitutively expressed *hsp70* genes, while the CY-B related homologs appear to represent the major heat-inducible *hsp70*s in *P. tetraurelia* as well. This result suggests conserved functions and a general expression pattern of the *Paramecium* Hsp70 gene family, at least between *P. caudatum* and *P. tetraurelia*. In this context it is interesting to note that *Tetrahymena* features also constitutively expressed and heat-inducible CY-*hsp70*s [32,45], which form together with the single *Ichthyophthirius multifiliis* CY-*hsp70* homolog a sister clade to all *Paramecium* CY-homologs (Figure 2). In this cytosolic *Ichthyophthirius–Tetrahymena* clade, the constitutively expressed *T. thermophila hsp70-4* gene clusters together with orthologs of *T. malaccensis*, *T. borealis* and *I. multifiliis* by forming a sister clade to all other *Tetrahymena* CY-homologs including the heat-inducible *T. thermophila hsp70-2* gene. Furthermore, while heat-inducible and constitutively expressed CY-*hsp70*s are common in higher eukaryotes too, they obviously do not form clear separated clades (cf. Additional file 4: Figure S2). In conjunction with our findings for ciliate *hsp70*s, this strongly indicates that heat-inducibility in cytosolic Hsp70s evolved several times independently during eukaryote evolution; at least twice within closely related unicellular eukaryotes and within metazoans. This result provides a striking example of convergent evolution during subfunctionalization among eukaryotes.

Our relative expression analyses also demonstrated that the *P. caudatum* Hsp70-groups ER-A and ER-B showed comparatively similar transcription levels at optimum temperatures, but the relative amount was considerably lower compared to the major constitutively expressed Hsp70-group CY-A. Heat treating the *P. caudatum* cells significantly induced the mRNA expression of both ER-type groups, but only to a comparatively small amount of 2.0-fold for ER-A and 2.7-fold for ER-B. Our study, therefore, partially supports the findings of Hori and Fujishima [46], who showed only trace amounts of ER-type *hsp70* mRNA when *P. caudatum* cells were cultured at 25°C, but also an up-regulation of approx. 4-fold when cells were heat shocked at 35°C. On the other hand, they suggested a predominant expression of ER-type *hsp70*s because they could not detect cytosolic homologs [46]. This is in contrast to our results showing that the cytosolic Hsp70-group CY-A encompassed the major constitutively expressed *hsp70*s, while group CY-B covered the major inducible homologs. Further, the relative mRNA levels of the ER-groups were nearly 23% less of the relative cytosolic mRNA transcript amount at optimum temperatures. And after heat stress, the cytosolic *hsp70* transcription level was about 2.5-fold higher than that of all ER-type homologs, mainly because of the striking induction of the CY-B group, but also due to the significant up-regulation of the CY-A group *hsp70*s. Nevertheless, studies on other eukaryotic organisms revealed that ER-type Hsp70 proteins, mostly designated as GRP78 or BiP, are abundant proteins in animal cells as well [47]. They are primarily induced by glucose depletion [48], and also due to cadmium exposure [49], during apoptosis [50] or due to the presence of unfolded or misfolded proteins in the ER. However, they are not significantly heat-inducible [51], while *P. caudatum* ER-type *hsp70*s seem to be. Therefore, experiments on the gene expression of these ER-type *hsp70* genes investigating the effect of endosymbionts [46,52] or other stressors would be valuable in unveiling their role in the stress response of *Paramecium.*

In this study, we further identified one *hsp70* gene encoding for a mitochondrial Hsp70 protein (mtHsp70). These proteins are required for the translocation of cytosolic preproteins across the mitochondrial membrane as well as the subsequent folding reactions in the mitochondrial matrix [53,54]. In most organisms, organelle-specific Hsp70s are generally encoded by a single gene [55], but in *Saccharomyces cerevisiae* three distinct *mtHsp70* genes are described, and *P. tetraurelia* seems to have at least two functional *mthsp70*s. Humans seemingly hold only one chaperone-active mtHsp70 protein (HSPA9B), while another highly similar protein (HSPA9A) plays a major role in the import of preproteins into the mitochondria. The single *mtHsp70* gene *PcHsp70MT1a* that we have observed in *P. caudatum* showed a constitutive expression pattern (Figure 3A), suggesting its essential role for mitochondria to function properly under normal physiological conditions. The 1.7-fold up-regulation in mRNA levels after heat shock, which is comparable to *S. cerevisiae* or *Blastocladiella emersonii mtHsp70*s [27,53], implies also its key role for proper mitochondrial function under heat stress. Though elevated temperatures can lead to enhanced oxidative stress, this might be overcome by an increased protein import into the mitochondria or an enhanced chaperone activity. Whether the *mthsp70* gene in *P. caudatum* covers both chaperone- and preprotein translocation activity and whether the *P. tetraurelia mthsp70*s have undergone subfunctionalization after gene duplication should be determined by future studies addressing Hsp70 protein interactions.

The phylogenetic and evolutionary patterns detected within the *Paramecium* Hsp70 gene family support the model of divergent evolution, while the different Hsp70-groups seem to have evolved under functional and structural constraints. On the other hand, in relation to the further investigated ciliate species and metazoans, these patterns indicate distinct evolutionary pathways and convergent evolution (cf. Figure 2). Therefore, this multigene family seems to be differentially affected also in ciliates by gene duplication events and/or whole genome duplications, by gene loss and retention, subfunctionalization and/or pseudogenization. The finding of a similar basal gene duplication pattern of the CY- and the ER-type *hsp70*s in *Paramecium*, for example, is indicative of a whole-genome duplication (WGD) event within the last common ancestor of *P. caudatum* and *P. tetraurelia*. It also may point to an intermediary WGD that occurred within the genus *Paramecium*, because the most recent WGD is supposed to coincide with rapid speciation events that gave rise to the *P. ‘aurelia’* complex (cf. Figure five in [31]). Further, an old WGD is thought to have occurred before the divergence of *Paramecium* and *Tetrahymena*[31], but is still under question due to recent studies that failed to detect evidence for WGD in *T. thermophila* and on the *Ichthyophthirius*/*Tetrahymena* branch [56,57].

The age of these WGD events would be of major significance to clarify speciation and radiation events in ciliates as well as to understand the dynamics of gene loss and retention or the metabolic adaptation after a WGD [58]. For example, the dates of these duplications could be interesting in consideration of the phylogenetic relationships among the genus *Paramecium*, whether these events occurred within the last common *Paramecium* ancestor or before/after certain speciation events. Insights into the different scenarios would be of importance to potentially support the proposed subdivision of the genus *Paramecium* into the four subgenera *Chloroparamecium*, *Helianter*, *Cypriostomum* and *Paramecium*[59]. Here, further analyses of the Hsp70 multigene family of at least three additional *Paramecium* species (e.g., *Paramecium* [*Chloroparamecium*] *bursaria*, *Paramecium* [*Helianter*] *putrinum* and *Paramecium* [*Cypriostomum*] *calkinsi*) would be required. However, due to a missing reliable molecular clock for ciliate divergence times (e.g. approx. 1500 to 600 Ma for *Paramecium*/*Tetrahymena* divergence [60,61]) and recent findings on *P. tetraurelia* that indicate extraordinarily reduced DNA mutation rates [37], more genome data would be favourable to estimate the dates of *Paramecium* species divergence or the timing of WGD events in the ciliate phylum.

Assuming a WGD event within the last common ancestor of *P. caudatum* and *P. tetraurelia*, the lack of MT paralogs in *P. caudatum* has to be the result of pseudogenization and a rapid gene loss. Even though many of the duplicates of the recent WGD in *P. tetraurelia* are functionally redundant and are supposed to be progressively lost, most of the gene duplicates did not go through such a rapid elimination [31]. On the other hand, the four putative *mthsp70*s in *P. tetraurelia*, which seem to comprise two functional genes and two pseudogenes, are suggestive of an intermediary WGD event causing the Hsp70-group differentiation in *Paramecium*. Since our study on *P. caudatum* was based on cDNA and all the detected homologs show typical Hsp70 family signatures and motifs as well as no premature stop codons or frameshifts, they can be considered as functional *hsp70* genes. Therefore, we have not detected either potentially expressed or unexpressed *hsp70* pseudogenes, which would represent a hallmark of the birth-and-death process after duplication events. Hence, to disentangle in more detail the different patterns of concerted and non-concerted evolution that shaped the *P. caudatum* Hsp70 gene family, further gDNA/genome analyses would be necessary to unveil potential *hsp70* pseudogenes in *P. caudatum*. Moreover, comparative genome analyses can uncover the gene order and orientation of recent *hsp70*s to infer pre-duplication states and to predict patterns of duplicate loss and retention. Such analyses can further improve our understanding of the evolution of such gene families in *Paramecium* and other microbial eukaryotes.

## Conclusions

The results of this study demonstrate first, that the Hsp70 multigene family of *Paramecium caudatum* comprises several homologous genes that can be assigned to three major Hsp70-subfamilies: one mitochondrial, five cytosolic and five endoplasmic-reticulum-related *hsp70*s. Phylogenetic analyses further revealed two separate Hsp70-groups within the cytosolic as well as the ER-type homologs, showing a closer relationship to *Paramecium tetraurelia* orthologs than to each other. These groups are derivatives of duplication events preceding speciation and have evolved in a conservative fashion under purifying selection. Second, our results revealed significant differences in the expression levels of these genes as well as significant heat-shock effects. While we observed constitutively expressed *hsp70* genes for all three cellular compartments, indicating the general importance of these proteins, our analyses also unveiled a major inducible Hsp70-group. This group was assigned as a cytosolic member with a low basal expression level, but a striking up-regulation of approx. 84-fold after heat shock. A similar expression pattern could be found in *P. tetraurelia* via the analyses of different EST libraries, which suggests conserved functions of orthologous *hsp70*s, at least between these two *Paramecium* species. On the other hand, the constitutively expressed and heat-induced cytosol-type *hsp70*s evolved independently in *Paramecium* and in its close relative *Tetrahymena*, but also in higher eukaryotes. This fact provides evidence of convergent evolution during subfunctionalization of eukaryote *hsp70*s. In addition, the herein developed RT-qPCR assay provides a crucial tool for future selective studies examining the effect of several stresses on the *hsp70* gene expression in *Paramecium*, a complex unicellular eukaryote that is an excellent model organism in cellular physiology, developmental biology, genome evolution and epigenetic inheritance.

## Methods

### *Paramecium* stock and culture conditions

The *Paramecium caudatum* stock GR*L*-1, isolated from a freshwater sample of a natural habitat in Greece, Livadia, was grown in a modified 0.25% Cerophyl infusion [62] inoculated with *Pseudomonas fluorescens*, as described previously [63]. The clonal stock culture was initiated from a single *Paramecium* cell to minimize locus heterozygosity. Wheat Grass Powder, used as the nutrient supply for the Cerophyl infusion, was purchased from GSE Vertrieb GmbH. The *P. fluorescens* strain SBW25 EeZY-6KX [64] was acquired from the University of Oxford. Cultures were maintained in microprocessor-controlled, cooled incubators obtained from BINDER GmbH (Type KB 53).

### RNA isolation and cDNA preparation

RNA was isolated from 2 ml paramecia culture (~3500 cells ml^-1^) maintained at 25°C, and from 2 ml culture heat shocked for one hour at 35°C. Total RNA was prepared using the acid phenol-guanidinium thiocyanate procedure [65] with commercial TRIzol® Reagent (Invitrogen Life Technologies) as specified in the manufacturer’s protocol. The purified RNA was treated with RNase-free TURBO™ DNase (Ambion® Inc.), which was subsequently removed with DNase Inactivation Reagent (Ambion® Inc.) following the manufacturer’s protocol. The concentration and purity of total RNA was determined by absorption at OD_260_, OD_260/280_, OD_260/230_ and by gel electrophoresis. cDNA synthesis was carried out according to the instructions given in the SuperScript® III Reverse Transcriptase protocol (Invitrogen Life Technologies) using oligo(dT)_18_ primers (Fermentas Life Sciences).

### PCR amplification and cDNA-library construction

Degenerated primers were designed for conserved regions of the Hsp70 family on the basis of an amino acid as well as a sequence alignment including *hsp70* homologous genes of three major Hsp70-subfamilies (cytosol, endoplasmic reticulum, mitochondria) from different eukaryotes. The forward primer Hsp70ForDeg corresponded to the amino acid sequence IGIDLGT: 5^′^-ATW GGH ATH GAY TTR GGW AC-3^′^ and the reverse primer Hsp70RevDeg corresponded to the amino acid sequence PQIEVTF: 5^′^-TCR AAD GWR ACT TCR ATT TRW GG-3^′^. The amplification was performed in a final volume of 50 μl containing 20 ng cDNA, 1 mM of each primer, 1 U Phusion™ High-Fidelity DNA Polymerase (New England Biolabs Inc.), 1 × Phusion™ HF Buffer with 2 mM MgCl_2_, 200 μM of each dNTP and 1.5 μl DMSO-d_6_ (Sigma-Aldrich®). PCR conditions were as follows: one minute initial denaturation (98°C); 35 cycles of fifteen seconds at 98°C, thirty seconds at 50°C and fifty seconds at 72°C; and a final extension step of seven minutes at 72°C. PCR products were visualised using agarose gel electrophoresis with ethidium bromide staining and bands of the expected size (~1.6 kb) were excised. DNA was extracted using the NucleoSpin® Extract II kit following the manufacturer’s protocol. The purified DNA fragments were tailed by adding a 3^′^ terminal ‘A’ overhang onto the PCR products using a non-proofreading *Taq* polymerase (Fermentas Life Sciences) and subsequently ligated into a pGEM®-T Vector (Promega GmbH) according to the manufacturer’s instructions. After transformation of competent *E. coli* JM109 cells, sixty of more than 200 clones that contained the expected insert size were sequenced with standard vector primers using the BigDye® Terminator v3.1 Cycle Sequencing Kit (Applied Biosystems). Sequencing reactions were performed and analyzed in both directions and with specifically designed internal sequencing primers for full-length sequencing on an ABI 3100 Genetic Analyzer (Applied Biosystems).

To determine the levels of differential gene expression of the diverse *hsp70* homologs within subsequent RT-qPCR runs, we further amplified two potential reference genes: the glyceraldehyde-3-phosphate dehydrogenase gene (*GAPDH*) and the elongation factor-1 alpha gene (*EF-1α*). Degenerated oligonucleotides, targeted to conserved regions of known *GAPDH* and *EF-1α* gene sequences from *Paramecium tetraurelia*, *Tetrahymena thermophila* and *T. pyriformis,* were used as primers to amplify the respective *P. caudatum* genes. The primers GAPDHFor1: 5^′^-GGT AGA TTR GTW TTR AGA GC-3^′^ and GAPDHRev: 5^′^-CCS CAY TCR TTR TCR TAC CA-3^′^ amplified a ~1kb fragment, and the primers EF1aFor_Deg: 5^′^-GGW AAR TCH ACY WCH WSH GGT CAC-3^′^ and EF1aRev_Deg: 5^′^-GCR ACS RYT WVY TTC ATR TCT C-3^′^ produced a ~1.4 kb fragment. Amplification and subsequent procedures were carried out according to the Hsp70 methods as described above with changes in the annealing temperatures of 56°C for *GAPDH* and 53°C for *EF-1α*. Furthermore, ten clones of the respective genes were sequenced to estimate the intra-individual sequence divergence.

### cDNA sequence analyses and phylogenetic reconstruction

Sequence data were assembled using the programme Vector NTI v.10.1 (Invitrogen Corp.) and compared with the non-redundant sequence database using NCBI-BLAST to assign the *hsp70* sequences to the three Hsp70-subfamilies: cytosol, ER and mitochondria. Sequence statistics, calculation of synonymous (*d*_*S*_) and non-synonymous (*d*_*N*_) site differences, and codon-based Z-tests using the Nei-Gojobori model [39] to estimate selective pressures were carried out with MEGA5 [66]. The automated GENECONV algorithm [67,68] as implemented in the Recombination Detection Program (RDP4 Beta 4.18 [69]) was applied to detect potential gene conversion events between the different *hsp70* homologs using the auto-mask option. Motif analyses were done with InterProScan [70] implemented in Geneious Pro v.5.4.2, which was also used for sequence logo calculations [71]. The *P. caudatum* Hsp70 amino acid sequences were aligned to homologous genes from different prokaryotes and eukaryotes available in GenBank, or derived from several genome databases (see Additional file 5: Table S3 for a complete list of accession numbers, descriptions and the sources used) using Probalign v.1.3 [72]. The final alignment contained 97 taxa and was manually trimmed to the length of *P. caudatum* Hsp70s, resulting in an overall length of 489 amino acids including gaps. The programme ProtTest v.3.0 [73] was used to find the most appropriate amino acid substitution model of the Hsp70 protein evolution required for Maximum-Likelihood (ML) calculations and Bayesian analysis (BA). The best-fit according to AICc was reported for the LG model [74] with I = 0.084 and *Γ* = 0.965. Maximum likelihood analyses were conducted with RAxML v.7.3.0 [75,76] using the PROTGAMMALG model. The statistical robustness of the nodes of the inferred best-scoring ML tree was determined by 1,000 rapid bootstrap replicates. The Bayesian analysis was performed with MrBayes v.3.1.2 [77] that was customized to use the LG+I+*Γ* model. We carried out two independent runs for 2,000,000 generations, with a sampling frequency of 1,000 generations. Each run used four chains, one cold and three heated. From the sampled trees, we discarded the first 25% as burn-in, ensuring stable likelihood values. The remaining trees were used to compute the consensus tree. The programmes Probalign and the fast hybrid versions of RAxML and MrBayes were run on the CIPRES Science Gateway v.3.3 server [78]. The resulting phylogenetic trees were visualized and arranged for publication using FigTree v.1.3.1 and Corel Draw X4.

### Reverse transcription quantitative real-time PCR

RNA was extracted from 3 × 1 ml *P. caudatum* culture (~750 cells ml^-1^) maintained at 28°C as the control treatment (optimum temperature) and from paramecia that were heat shocked for two hours at 34°C. The length of the heat treatment was chosen because of the results of a previous time-course experiment (1 h, 2 h and 4 h) revealing this duration as an appropriate value with comparatively high *hsp70* gene induction and no *Paramecium* mortality (data not shown). The respective treatment temperatures were set to values around the previously determined optimum and maximum growth temperatures of *P. caudatum*[79]. The experiment was run with five biological replicates. RNA was prepared using RNeasy Mini spin columns (QIAGEN) following the manufacturer’s protocol for total RNA purification from animal cells. The buffer RLT was furnished with β-mercaptoethanol and pelleted paramecia cells from 1 ml culture were lysed for five minutes in 350 μl buffer RLT. One volume of 70% ethanol was added to each of the three corresponding lysates, which were consecutively transferred to the RNeasy Mini spin columns. All further steps were carried out according to the instructions, including the optional drying and the second elution step. Potentially leftover genomic DNA was digested by an RNase-free TURBO™ DNase (Ambion® Inc.) treatment. The DNase was subsequently removed with DNase Inactivation Reagent (Ambion® Inc.) following the manufacturer’s protocol. The RNA concentrations, purities and integrities of all preparations were determined by absorption at OD_260_, OD_260/280_ and OD_260/230_, by gel electrophoresis and with the Agilent 2100 Bioanalyzer™ (Agilent Technologies). The mean RNA yield per sample was 161±12 ng/μl (mean±SEM) and the RNA integrity number (RIN) for all specimens was ≥ 9.2 indicating high quality RNA [80]. For each replicate, 500 ng RNA was reverse transcribed into double stranded cDNA using the RevertAid™ H^-^ M-MuLV reverse transcriptase and a mixture of oligo(dT)_18_ and random hexamer primers (Fermentas Life Sciences) in a final volume of 20 μl according to the manufacturer’s instructions.

The differential mRNA expression levels for five different Hsp70-groups (CY-A, CY-B, ER-A, ER-B, and MT) were quantified with an RT-qPCR assay using an ABI PRISM® 7300 Sequence Detector System (Applied Biosystems). The two reference genes, *GAPDH* and *EF-1α*, were used for normalization. The target specific primers and MGB™ hydrolysis probes (Table 2, see also Additional file 6: Figure S3) were designed and validated according to Applied Biosystems relative quantification guidelines using Primer Express® v.2.0 (Applied Biosystems). Each sample of five biological replicates was analyzed in technical triplicates. Each 20 μl reaction contained 1 × reaction buffer (Invitrogen, Platinum® quantitative PCR Supermix-UDG with Rox), 200 μM of each dNTP, 900 nM of each target specific primer, 250 nM of the target specific probe, 5 mM MgCl_2_ and 10 ng cDNA (10 ng of reverse transcribed RNA), or 10 ng RNA within the –RT control, respectively. Thermal cycling conditions were as follows: two minutes at 50°C as the initial step to activate the uracil glycosylase (UNG), two minutes at 95°C to inactivate the UNG and as the initial denaturation step, followed by 40 cycles for fifteen seconds at 95°C and thirty seconds at 60°C.

**Table 2:** **Nucleotide sequences for the specific primers and MGB™ probes, melting temperatures (*T***_**m**_**), fragment lengths and PCR efficiencies**

**Target**	**Primer/Probe**	**Nucleotide sequence**	***T***_**m**_	**Fragment length**	**PCR efficiency**
*Hsp70* CY-A	Hsp70cyA_rt_F	5^′^-TCA GGA GCT GAT GAC AAA CCA A-3^′^	59°C	74 bp	90%
	Hsp70cyA_rt_R	5^′^-ATC TCC TCT GGA TGG AAT TTC TTG-3^′^	59°C		
	Hsp70cyA_MGB_VIC	5^′^-TGT GGT TAA GTA TAA AGG TGA G-3^′^	67°C		
*Hsp70* CY-B	Hsp70cyB_rt_F4	5^′^-TGC ATA TGG TGC TGC TGT TTA AG-3^′^	59°C	133 bp	89%
	Hsp70cyB_rt_R2	5^′^-TAA TAC ACT CAT TAC ACC ACC TGC AG-3^′^	59°C		
	Hsp70cyB_MGB_VIC	5^′^-CCA CTT TCA TTA GGA ATT-3^′^	68°C		
*Hsp70* ER-A	Hsp70ERA_rt_F	5^′^-GAA GTT CTC ACT AGA GCC AGA TTC G-3^′^	59°C	76 bp	93%
	Hsp70ERA_rt_R	5^′^-CTG ATT ACA TGG GAC CAG TTG TCT-3^′^	58°C		
	Hsp70ERA_MGB_VIC	5^′^-ACT CAA TTC AGA TCT C-3^′^	68°C		
*Hsp70* ER-B	Hsp70ERB_rt_F	5^′^-GTT GAG AAG GGA ACC TAA TAA AAG GTC-3^′^	59°C	98 bp	92%
	Hsp70ERB_rt_R	5^′^-GTC CCA AAT ATC CTT CAG CAA TCT-3^′^	59°C		
	Hsp70ERB_MGB_VIC	5^′^-CAG AGG AGA TTA GTG CCA TG-3^′^	68°C		
*Hsp70* MT	Hsp70mt_rt_F	5^′^-ACC AGA ATG CCA AAA GTC CAA-3^′^	58°C	76 bp	91%
	Hsp70mt_rt_R	5^′^-CAT CTG GGT TGA CTG ACT TGT TG-3^′^	58°C		
	Hsp70mt_MGB_VIC	5^′^-AAG ATT TAT TCG ACA AAC CA-3^′^	67°C		
*GAPDH*	GAPDH_rt_F	5^′^-GCT GCC AAG GCT GTT GGT-3^′^	58°C	75 bp	92%
	GAPDH_rt_R	5^′^-TGT TGG AAC TCT GAA GGC CAT AC-3^′^	59°C		
	GAPDH_MGB_FAM	5^′^-TCC CAG AAA TCA AAG-3^′^	67°C		
*EF-1a*	EF1a_rtF2	5^′^-TTG ATG CCC CAG GAC ATA GAG-3^′^	59°C	95 bp	94%
	EF1a_rtR2	5^′^-TCC TGC TGG TGA GGC AAT C-3^′^	60°C		
	EF1a_MGB_FAM	5^′^-TAC AGG AAC ATC ATA AGC-3^′^	68°C		

### Statistical analyses of RT-qPCR

The stability of the two reference genes *GAPDH* and *EF-1α* were evaluated with the geNorm v.3.5 applet [40]. The amplification efficiency-based method of the programme REST 2009 v.2.0.13 [81] was used for gene normalization and quantification. The significance of the differences between the controls (28°C) and the heat-shock treatments (34°C) was determined with a strong randomization (iteration) test as implemented in the programme. The test was performed with 10,000 random reallocations of samples and controls between the groups by counting the number of times the relative expression on the randomly assigned group was greater than the sample data (REST 2009, software manual).

### Comparative EST library screening

To unveil if the detected differential *hsp70* gene expression of *P. caudatum* is conserved among *Paramecium*, two EST libraries of *P. tetraurelia* were screened for specific gene sequences. The EST libraries were constructed at Genoscope - Centre National de Séquençage, France. Both libraries were created from total RNA of vegetative cells, using the CloneMiner cDNA library construction kit (Invitrogen). The first library was derived from *P. tetraurelia* cells grown at 27°C (NCBI dbEST ID 20983) and the second library from cells heat treated at 35°C (NCBI dbEST ID 20987). Both libraries were screened for orthologous EST sequences of the five *P. caudatum* Hsp70-groups using following *P. tetraurelia* nucleotide sequences (see GenBank accession numbers) as references: CY-A = CR932269 and CR933372; CY-B = CR933371, CR933370 and CR933369; ER-A = CR932268 and CR932267, ER-B = CR932266, MT = AF031366 and XM_001461342.1. Screening was performed using the Local Blast engine in Bioedit v.7.0.9.0 [82] with an e-value cut-off of e < E^-100^. EST counts for each *P. tetraurelia* ortholog were used to calculate an approximate expression level of the five Hsp70-groups quoted in transcripts per million.

## Competing interests

The authors declare that they have no competing interests.

## Authors’ contributions

SK participated in the study design, carried out the molecular, experimental and phylogenetic investigations, performed the statistical analyses and drafted the manuscript. MS contributed to the discussion and interpretation of the results and manuscript preparation. TUB conceived of this study, participated in its design and coordination and manuscript preparation. All authors read and approved the final manuscript.

## Supplementary Material

Additional file 1: Table S1 Pairwise evolutionary distances between *P. caudatum* Hsp70 sequences. (PDF 23 kb) Click here for file

Additional file 2: Figure S1 *Paramecium caudatum* Hsp70 amino acid alignment with indicated motifs and family signatures. Click here for file

Additional file 4: Figure S2 Uncollapsed Maximum-Likelihood tree of pro- and eukaryotic Hsp70s. Click here for file

Additional file 3: Table S2 Codon-based test of purifying selection for analysis between *P. caudatum* Hsp70 sequences. Click here for file

Additional file 5: Table S3 List of accession numbers, descriptions and sources used for phylogenetic analyses. Click here for file

Additional file 6: Figure S3 *Paramecium caudatum* Hsp70 nucleotide sequence alignment with indicated binding sites for primer and MGB™ TaqMan® hydrolysis probes. Click here for file
