# Supplementary material for: Convergent evolution of heat-inducibility during subfunctionalization of the Hsp70 gene family
Source: BMC Evol Biol. 2013 Feb 21;13:49. doi: 10.1186/1471-2148-13-49 (PMC3606833; doi:10.1186/1471-2148-13-49)
Supplement: Additional file 1: Table S1 — Pairwise evolutionary distances between P. caudatum Hsp70 sequences. (PDF 23 kb) [file 1471-2148-13-49-S1.pdf]

Table S1

Pairwise evolutionary distances between *P. caudatum* Hsp70 sequences

	Paramecium caudatum PcHsp70										
	CY1a	CY1b	CY1c	CY2a	CY2b	ER1a	ER1b	ER2a	ER2b	ER2c	MT1a
PcHsp70											
CY1a		0	2	66	82	185	185	198	198	198	203
CY1b	4		2	66	82	185	185	198	198	198	203
CY1c	11	9		67	83	185	185	198	198	198	203
CY2a	342	340	337		23	180	180	186	186	186	202
CY2b	363	361	358	35		183	183	188	188	188	215
ER1a	485	485	486	468	470		0	87	87	87	217
ER1b	485	485	486	468	470	1		87	87	87	217
ER2a	512	512	510	484	492	317	318		0	0	224
ER2b	512	512	510	484	492	316	317	3		0	224
ER2c	511	511	509	483	491	316	317	2	1		224
MT1a	524	521	525	487	498	543	544	560	560	560	

The number of base differences (below diagonal) and amino acid differences (above diagonal) per sequence for comparisons of eleven *P. caudatum hsp70* homologs are shown. Codon positions included were 1st+2nd+3rd+Noncoding. All ambiguous positions were removed for each sequence pair with a total of 1395 bp or 465 aa positions, respectively, in the final datasets. Analyses were conducted in MEGA5.
