# Supplementary material for: Convergent evolution of heat-inducibility during subfunctionalization of the Hsp70 gene family
Source: BMC Evol Biol. 2013 Feb 21;13:49. doi: 10.1186/1471-2148-13-49 (PMC3606833; doi:10.1186/1471-2148-13-49)
Supplement: Additional file 2: Figure S1 — Paramecium caudatum Hsp70 amino acid alignment with indicated motifs and family signatures. [file 1471-2148-13-49-S2.pdf]

```
                      10        20        30        40        50        60        70        80
                       |         |         |         |         |         |         |         |
PcHsp70CY1a  TYSCVGVWINDKVEIIPNDQGNRTTPSYVGFT-DTERLIGDAAKNQVARNPTNTVFDAKRLIGRKFADNTVQKDTKLWPF 79
PcHsp70CY1b  TYSCVGVWINDKVEIIPNDQGNRTTPSYVGFT-DTERLIGDAAKNQVARNPTNTVFDAKRLIGRKFADNTVQKDTKLWPF 79
PcHsp70CY1c  TYSCVGVWINDKVEIIPNDQGNRTTPSYVGFT-DTERLIGDAAKNQVARNPTNTVFDAKRLIGRKFADNTVQKDTKLWPF 79
PcHsp70CY2a  TYSCVGVFINDKVEIISNDQGNRTTPSYVAFT-DGERLIGDAAKNQVARNPNNTVFDAKRLIGRKFNEQTVQKDIKLWPF 79
PcHsp70CY2b  SYSCVGVFINDKVEIISNDQGKRTTPSYVAFT-DGERLIGDAAKNQVARNPNNTVFDAKRLIGRKFNEQTVQKDIKLWPF 79
PcHsp70ER1a  TYSCVGIYKNGNVEIIPNEQGNRITPSVVAFT-DEERLIGEAAKNQAAINPTRTLYDVKRLIGRKYTDQTIQYDKKFMPY 79
PcHsp70ER1b  TYSCVGIYKNGNVEIIPNEQGNRITPSVVAFT-DEERLIGEAAKNQAAINPTRTLYDVKRLIGRKYTDQTIQYDKKFMPY 79
PcHsp70ER2a  TFSCVGVFRQGHVEIIPNELGNRITPSVVSFT-DSERLIGEAAKNQAATNPTRTLYDVKRLIGRKFTDSTIQYDRKFLPF 79
PcHsp70ER2b  TFSCVGVFRQGHVEIIPNELGNRITPSVVSFT-DSERLIGEAAKNQAATNPTRTLYDVKRLIGRKFTDSTIQYDRKFLPF 79
PcHsp70ER2c  TFSCVGVFRQGHVEIIPNELGNRITPSVVSFT-DSERLIGEAAKNQAATNPTRTLYDVKRLIGRKFTDSTIQYDRKFLPF 79
PcHsp70MT1a  TNSCVSIMESGTPKVIENAEGMRTTPSVVAFTADGQRIVGAPAKRQAVTNPENTVYATKRLIGRRFDDPNVQKDIKHLSY 80

                      90       100       110       120       130       140       150       160
                       |         |         |         |         |         |         |         |
PcHsp70CY1a  KVESGADDKPMIVVK-YKG-ETKKFHPEEISSMVLTKMKETAEAYLSKTVTKAVITVPAYFNDSQRQATKDAGAISGMNV 157
PcHsp70CY1b  KVESGADDKPMIVVK-YKG-ETKKFHPEEISSMVLTKMKETAEAYLSKTVTKAVITVPAYFNDSQRQATKDAGAISGMNV 157
PcHsp70CY1c  KVESGADDKPMIVVK-YKG-ETKKFHPEEISSMVLTKMKETAEAYLSKTVTKAVITVPAYFNDSQRQATKDAGAISGMNV 157
PcHsp70CY2a  KVEPGPDDKPLIVVK-FKG-ETKKFHPEEISSMVLIKMKEIAEAYLGKTVKNAVITVPAYFNDSQRQATKDAGAIAGLNV 157
PcHsp70CY2b  KVEPGPDDKPLIVVK-FKG-ETKKFHPEEISSMVLIKMKEIAEAYLGKTVKNAVITVPAYFNDSQRKATKDAGLIAGLNV 157
PcHsp70ER1a  DI-IDKDTKPYIKVTNIKGHQSKIFAPEEISAMVLTKMKEISETYLGKKVINAVVTVPAYFNDAQRQATKDAGTISGLNV 158
PcHsp70ER1b  DI-IDKDTKPYIKVTNIKGHQSKIFAPEEISAMVLTKMKEISETYLGKKVINAVVTVPAYFNDAQRQATKDAGTISGLNV 158
PcHsp70ER2a  EI-VDRDGKPYIQVE--KGTQQKVFAPEEISAMVLIKMKEIAEGYLGQTIVNAVVTVPAYFNDAQRQATKDAGQIAGLNV 156
PcHsp70ER2b  EI-VDRDGKPYIQVE--KGTQQKVFAPEEISAMVLIKMKEIAEGYLGQTIVNAVVTVPAYFNDAQRQATKDAGQIAGLNV 156
PcHsp70ER2c  EI-VDRDGKPYIQVE--KGTQQKVFAPEEISAMVLIKMKEIAEGYLGQTIVNAVVTVPAYFNDAQRQATKDAGQIAGLNV 156
PcHsp70MT1a  SVVKAQNGDAWVSLK--SG-QT--YSPSQMGAFVLIKMKETADAYIGKPQTKAVVTVPAYFNDSQRQATKDAGKIAGLDV 155

                     170       180       190       200       210       220       230       240
                       |         |         |         |         |         |         |         |
PcHsp70CY1a  LRIINEPTAAAIAYGLDKKAKGEEHVLIFDLGGGTFDVSLLAIEDGVFEVKATAGDTHLGGEDFDNKLVEYCCAEFMKKK 237
PcHsp70CY1b  LRIINEPTAAAIAYGLDKKAKGEEHVLIFDLGGGTFDVSLLAIEDGVFEVKATAGDTHLGGEDFDNKLVEYCCAEFMKKK 237
PcHsp70CY1c  LRIINEPTAAAIAYGLDKKAKGEEHVLIFDLGGGTFDVSLLAIEDGVFEVKATAGDTHLGGEDFDNKLVEYCCAEFLKKK 237
PcHsp70CY2a  LRIINEPTAASIAYGLDSKLSGEKNVLIFDLGGGTFDVSLLTLDEGIFEVKATSGDTHLGGEDFDNRLVEYCCMEFQKRK 237
PcHsp70CY2b  LRIINEPTAASIAYGLDSKLSGEKNVLIFDLGGGTFDVSLLTIDEGIFEVKATSGDTHLGGEDFDNRLVEYYCMEFQKRK 237
PcHsp70ER1a  VRILNEPTAAAIAYGLDKK-DGEKNILVFDLGGGTFDVSILTIDNGVFEVIATSGDTHLGGEDFDQRIIDHFIKVIKKKH 237
PcHsp70ER1b  VRILNEPTAAAIAYGLDKK-DGEKNILVFDLGGGTFDVSILTIDNGVFEVIATSGDTHLGGEDFDQRIIDHFIKVIKKKH 237
PcHsp70ER2a  VRILNEPTAAAIAYGLDSK-EKESNILVFDLGGGTFDVSILTIDNGVFEVQSTSGDTHLGGEDFDQRLIDHFIKLVQKKY 235
PcHsp70ER2b  VRILNEPTAAAIAYGLDSK-EKESNILVFDLGGGTFDVSILTIDNGVFEVQSTSGDTHLGGEDFDQRLIDHFIKLVQKKY 235
PcHsp70ER2c  VRILNEPTAAAIAYGLDSK-EKESNILVFDLGGGTFDVSILTIDNGVFEVQSTSGDTHLGGEDFDQRLIDHFIKLVQKKY 235
PcHsp70MT1a  LRIINEPTAAALAFGLEKK--DNKIIAVYDLGGGTFDISILEINAGVFEVKATNGDTSCGGEDVDSILSQWISNEFKAQA 233

                     250       260       270       280       290       300       310       320
                       |         |         |         |         |         |         |         |
PcHsp70Cy1a  GIDIRQNPRSLRRLRTQCERAKRVLSSANQTTIEVDALGDN----EDFNSTITRAKFEELCMSMFKECIPPVEKVLKDSG 313
PcHsp70Cy1b  GIDIRQNPRSLRRLRTQCERAKRVLSSANQTTIEVDALGDN----EDFNSTITRAKFEELCMSMFKECIPPVEKVLKDSG 313
PcHsp70Cy1c  GIDIRQNPRSLRRLRTQCERAKRVLSSANQTTIEVDALGDN----EDFNSTITRAKFEELCMSMFKECIPPVEKVLKDSG 313
PcHsp70Cy2a  GVDIRSNNRALRRLRTQCERAKRILSSANQTTIELDALAEN----EDFNCQITRAKFEELCLDLFKKCIPPVEQVLKDSG 313
PcHsp70Cy2b  GVDIRSNNRALRRLRTQCERAKRILSAANQTTIELDALVEN----EDFNCQITRAKFEELCSDLFIKCIPPVQQVLKDSG 313
PcHsp70ER1a  NKDISADKRAIQKLKREVEKAKRALSATHETKIEIEDLVDG----LDFNEVLTRARFEELNSDLFKKTTGPMQSALEDSG 313
PcHsp70ER1b  NKDISADKRAIQKLKREVEKAKRALSATHETKIEIEDLVDG----LDFNEVLTRARFEELNSDLFKKTTGPMQSALEDSG 313
PcHsp70ER2a  NKDVSGDKRAIQKLKRESEKAKRRLSAAQEAKVEIEDLVEG----LDFSEVLTRAKFEELNSDLFRKTIEPMQTALNDSG 311
PcHsp70ER2b  NKDVSGDKRAIQKLKRESEKAKRRLSAAQEAKVEIEDLVEG----LDFSEVLTRAKFEELNSDLFRKTIEPMQTALNDSG 311
PcHsp70ER2c  NKDVSGDKRAIQKLKRESEKAKRRLSAAQEAKVEIEDLVEG----LDFSEVLTRAKFEELNSDLFRKTIEPMQTALNDSG 311
PcHsp70MT1a  GVDIQKDKMAVQRVREAAEKAKIELSSTTQTDINLPYLTADASGPKHCNLKLTRAKLESLTEDFLKKTVKPTENCIKDSG 313

                     330       340       350       360       370       380       390       400
                       |         |         |         |         |         |         |         |
PcHsp70Cy1a  ISKNQIHEVVLVGGSTRIPKVQELLRDYFNGKELNKSINPDEAVAYGAAVQAAILTGQGNEQVKDLLLLDVTPLSLGIET 393
PcHsp70Cy1b  ISKNQIHEVVLVGGSTRIPKVQELLRDYFNGKELNKSINPDEAVAYGAAVQAAILTGQGNEQVKDLLLLDVTPLSLGIET 393
PcHsp70Cy1c  ISKNQIHEVVLVGGSTRIPKVQDLLRDYFNGKELNKSINPDEAVAYGAAVQAAILTGQGNEQVKDLLLLDVTPLSLGIET 393
PcHsp70Cy2a  MSKNTIHEVVLVGGSTRIPKVQELLKDYFNGKELNKSINPDEAVAYGAAVQAAILTGTGSQKCENLVLLDVTPLSLGIET 393
PcHsp70Cy2b  MSKNTIHEVVLVGGSTRIPKVQELLKDYFNGKELNRSINPDEAVAYGAAVQAAIITGPGSQKCEKFQFLDVKPLSLGIET 393
PcHsp70ER1a  LKKNEVDEIVLVGGSSRIPKIRQIVKDFFNGKEANTGINPDEAVCYGAAIQGGIICGEESNETKGLIVIDATPLSLGIET 393
PcHsp70ER1b  LKKNEVDEIVLVGGSSRIPKIRQIVKDFFNGKEANTGINPDEAVCYGAAIQGGIICGEESNETKGLIVIDATPLSLGIET 393
PcHsp70ER2a  YKKSQIDEIVLVGGSSRIPKVRQIVKEFFEGKDPNTGINPDEAICYGAAIQGGLICGEKSEETDGLIVIDATPLSLGIET 391
PcHsp70ER2b  YKKSQIDEIVLVGGSSRIPKVRQIVKEFFEGKDPNTGINPDEAICYGAAIQGGLICGEKSEETDGLIVIDATPLSLGIET 391
PcHsp70ER2c  YKKSQIDEIVLVGGSSRIPKVRQIVKEFFEGKDPNTGINPDEAICYGAAIQGGLICGEKSEETDGLIVIDATPLSLGIET 391
PcHsp70MT1a  LDKSKIDEVILVGGMTRMPKVQKLVQDLFD-KPPNKSVNPDEAVSIGAAIQGGVLKG----DVKELLLLDVTPLSLGIET 388

                     410       420       430       440       450       460
                       |         |         |         |         |         |
PcHsp70Cy1a  AGGVMTVLIPRNTTIPTKKSQTFTTYADNQPGVLIQVYEGERQMTKDCHKLGQFNLDGIAPAPRGV 459
PcHsp70Cy1b  AGGVMTVLIPRNTTIPTKKSQTFTTYADNQPGVLIQVYEGERQMTKDCHKLGQFNLDGIAPAPRGV 459
PcHsp70Cy1c  AGGVMTVLIPRNTTIPTKKSQTFTTYADNQPGVLIQVYEGERQMTKDCHKLGQFNLDGIAPAPRGV 459
PcHsp70Cy2a  AGGVMSVLIPRNTTIPTKKSQVFSTYADNQPGVLIQVYEGERQMTKDCHKLGQFHLDGIAPAPRGV 459
PcHsp70Cy2b  AGGVMSVLIPRNTTIPTKKSQVFSTYADNQPGFLVQVYEGERQMTKDCHKLGEFHLDGIAPVPRGV 459
PcHsp70ER1a  VGGVMTKIIPKGSYIPTKKSQVFTTYQDQQQTVTISVFEGERPLVKDNHKLGTFDLTGIPPAPRGT 459
PcHsp70ER1b  VGGVMTKIIPKGSYIPTKKSQVFTTYQDQQQTVTISVFEGERPLVKDNHKLGTFDLTGIPPAPRGT 459
PcHsp70ER2a  VGGVMNKIIPKGSFIPTKKSQVFTTYVDNQQTVTITVFEGERPLVKDNHKLGQFDLTDIPKMPKGQ 457
PcHsp70ER2b  VGGVMNKIIPKGSFIPTKKSQVFTTYVDNQQTVTITVFEGERPLVKDNHKLGQFDLTDIPKMPKGQ 457
PcHsp70ER2c  VGGVMNKIIPKGSFIPTKKSQVFTTYVDNQQTVTITVFEGERPLVKDNHKLGQFDLTDIPKMPKGQ 457
PcHsp70MT1a  LGGVFTKMIPRNTTIPTKKSQTYSTASDNQTVVSIRVFQGEREMAADNKLLGQFDLSGIPPAPRGV 454
```

Figure S1. Multiple sequence alignment of the deduced amino acid sequences of *Paramecium caudatum* Hsp70s with specific features shaded in grey. The putative ATP/GTP-binding site motif A (P-loop) is underlined; the characteristic Hsp70 family signatures 2 and 3 are double-underlined; the putative bipartite nuclear localization signal is shown in a box with a dashed line frame and the potential signature for eukaryotic non-organellar Hsp70 proteins is shown in a solid line framed box. The Hsp70 abbreviations correspond to GenBank accession numbers as follows: PcHsp70CY1a - ADU54551, PcHsp70CY1b - ADU54552, PcHsp70CY1c - ADU54553, PcHsp70CY2a - ADU54554, PcHsp70CY2b - ADU54555, PcHsp70ER1a - ADU54556, PcHsp70ER1b - ADU54557, PcHsp70ER2a - ADU54558, PcHsp70ER2b - ADU54559, PcHsp70ER2c - ADU54560, PcHsp70MT1a - ADU54561.
