# Supplementary material for: Convergent evolution of heat-inducibility during subfunctionalization of the Hsp70 gene family
Source: BMC Evol Biol. 2013 Feb 21;13:49. doi: 10.1186/1471-2148-13-49 (PMC3606833; doi:10.1186/1471-2148-13-49)
Supplement: Additional file 4: Figure S2 — Uncollapsed Maximum-Likelihood tree of pro- and eukaryotic Hsp70s. [file 1471-2148-13-49-S4.pdf]

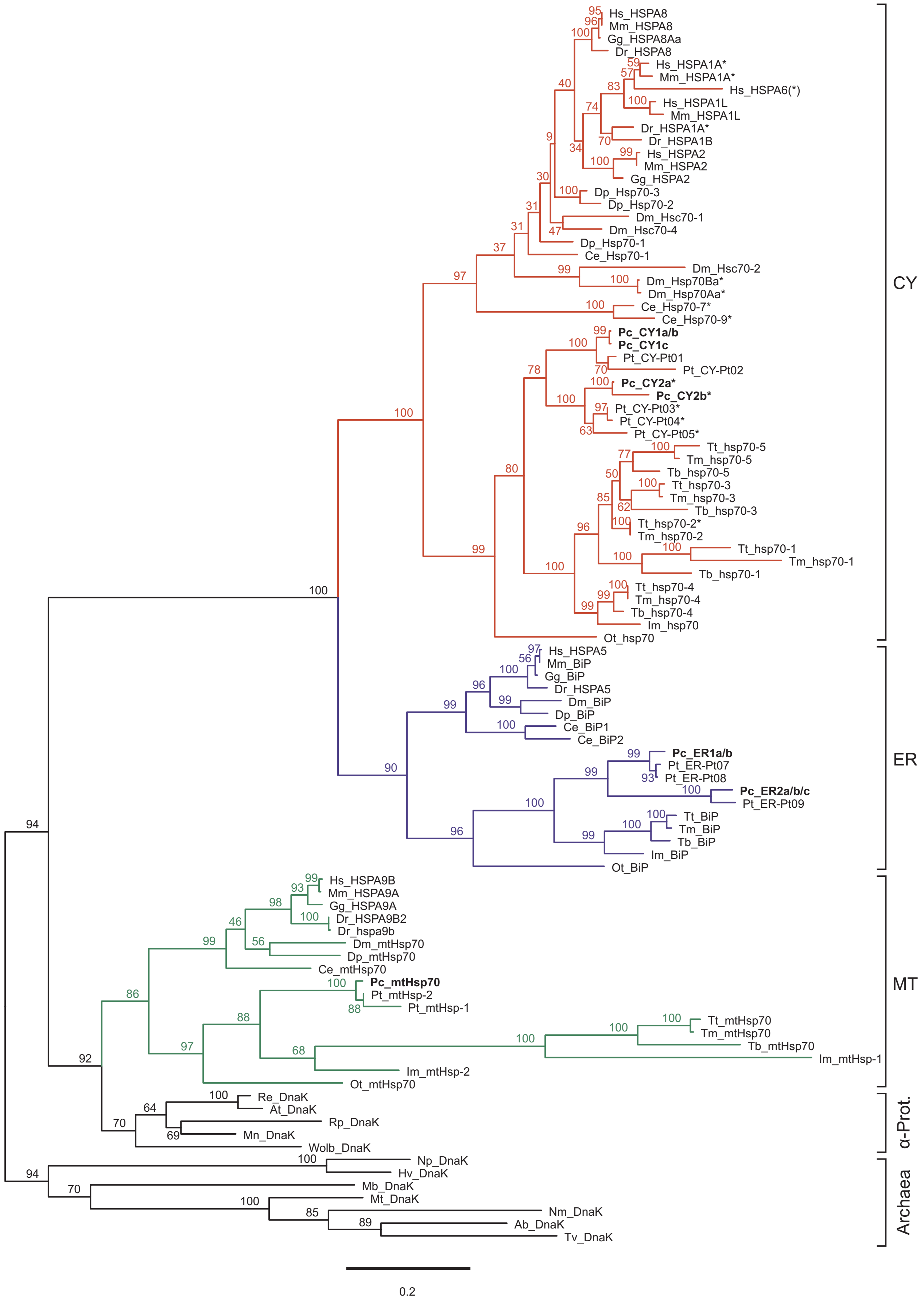

Figure S2. Maximum-Likelihood tree based on an amino acid alignment containing 97 homologous *hsp70* sequences from pro- and eukaryotes showing the typical Hsp70 family signatures. Sequences obtained within this study are shown in bold face. The alignment length was constricted to 489 amino acids including gaps. The LG+I+*Γ* protein evolution model with 1,000 rapid bootstrap replicates was used for the Maximum-Likelihood calculations. The Archaea DnaK sequences served as outgroup. Numbers at the nodes represent bootstrap support values. The three major Hsp70-subfamilies cytosol (CY), endoplasmic reticulum (ER) and mitochondria (MT) are indicated by squared brackets to the right. Highly heat inducible cytosol-type *hsp70* genes (if known) are specified by an asterisk. Tip labels correspond to the descriptions and GenBank accession numbers given in Additional file 5, Table S3. An electronic version is deposited in TreeBASE under accession number TB2:S13746.
