# Supplementary material for: Convergent evolution of heat-inducibility during subfunctionalization of the Hsp70 gene family
Source: BMC Evol Biol. 2013 Feb 21;13:49. doi: 10.1186/1471-2148-13-49 (PMC3606833; doi:10.1186/1471-2148-13-49)
Supplement: Additional file 3: Table S2 — Codon-based test of purifying selection for analysis between P. caudatum Hsp70 sequences. [file 1471-2148-13-49-S3.pdf]

Table S2

Codon-based test of purifying selection for analysis between *P. caudatum* Hsp70 sequences

	Paramecium caudatum PcHsp70										
	CY1a	CY1b	CY1c	CY2a	CY2b	ER1a	ER1b	ER2a	ER2b	ER2c	MT1a
PcHsp70											
CY1a		0.040	0.002	0.000	0.000	0.000	0.000	0.000	0.000	0.000	0.000
CY1b	0.020		0.009	0.000	0.000	0.000	0.000	0.000	0.000	0.000	0.000
CY1c	0.001	0.005		0.000	0.000	0.000	0.000	0.000	0.000	0.000	0.000
CY2a	0.000	0.000	0.000		0.494	0.000	0.000	0.000	0.000	0.000	0.000
CY2b	0.000	0.000	0.000	0.248		0.000	0.000	0.000	0.000	0.000	0.000
ER1a	0.000	0.000	0.000	0.000	0.000		0.299	0.000	0.000	0.000	0.000
ER1b	0.000	0.000	0.000	0.000	0.000	0.148		0.000	0.000	0.000	0.000
ER2a	0.000	0.000	0.000	0.000	0.000	0.000	0.000		0.074	0.148	0.000
ER2b	0.000	0.000	0.000	0.000	0.000	0.000	0.000	0.036		0.294	0.000
ER2c	0.000	0.000	0.000	0.000	0.000	0.000	0.000	0.069	0.151		0.000
MT1a	0.000	0.000	0.000	0.000	0.000	0.000	0.000	0.000	0.000	0.000	

The probability of rejecting the null hypothesis of strict-neutrality ($d_N = d_S$) (above diagonal) in favor of the alternative hypothesis of purifying selection ($d_N < d_S$) (below diagonal) is shown; d_S and d_N are the numbers of synonymous and nonsynonymous substitutions per site, respectively. The analyses were conducted using the Nei-Gojobori method and involved 11 nucleotide sequences. All ambiguous positions were removed for each sequence pair with a total of 465 positions in the final dataset. Analyses were conducted in MEGA5.
