# Supplementary figures and images for: Convergent evolution of heat-inducibility during subfunctionalization of the Hsp70 gene family
Source: BMC Evol Biol. 2013 Feb 21;13:49. doi: 10.1186/1471-2148-13-49 (PMC3606833; doi:10.1186/1471-2148-13-49)

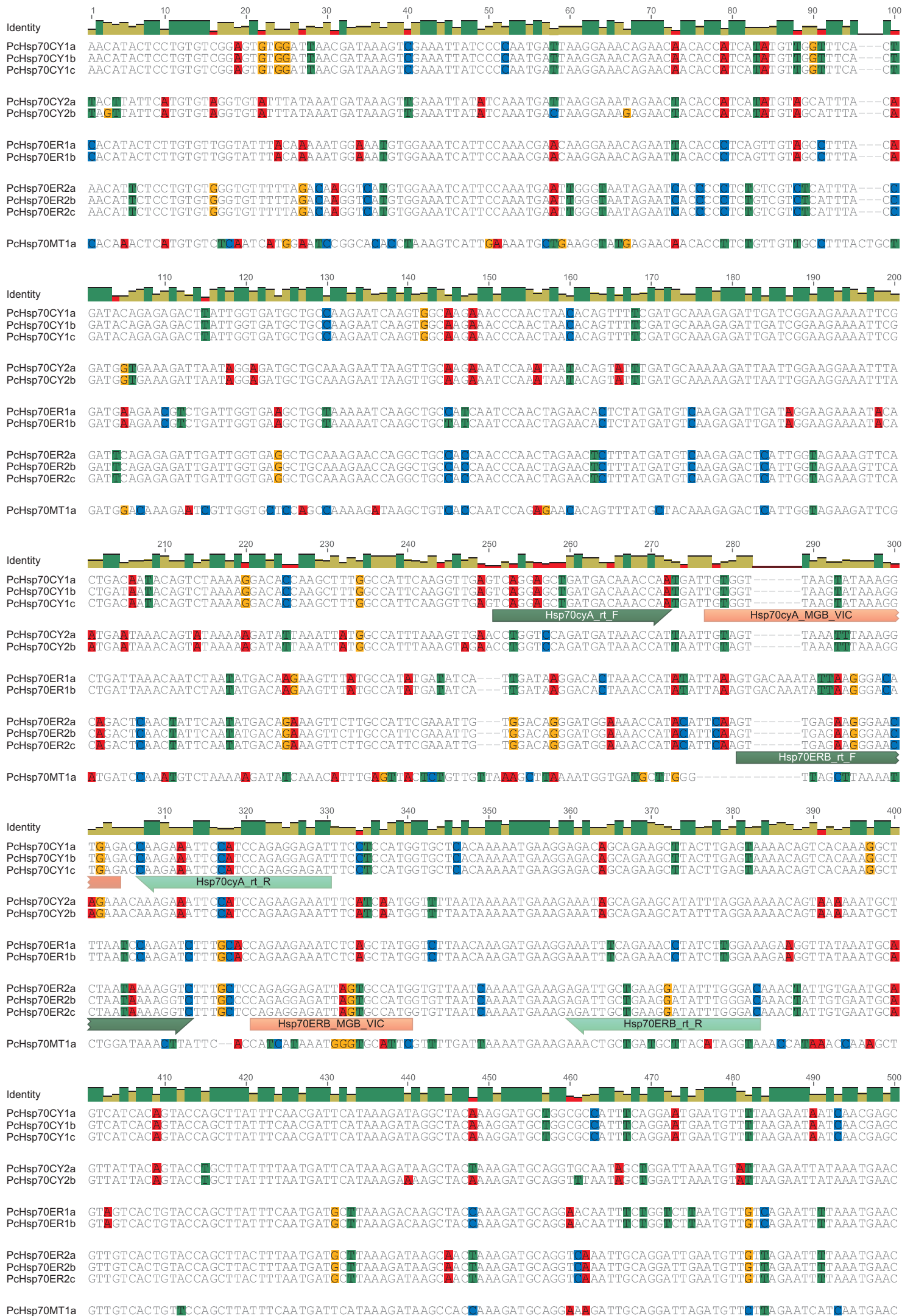

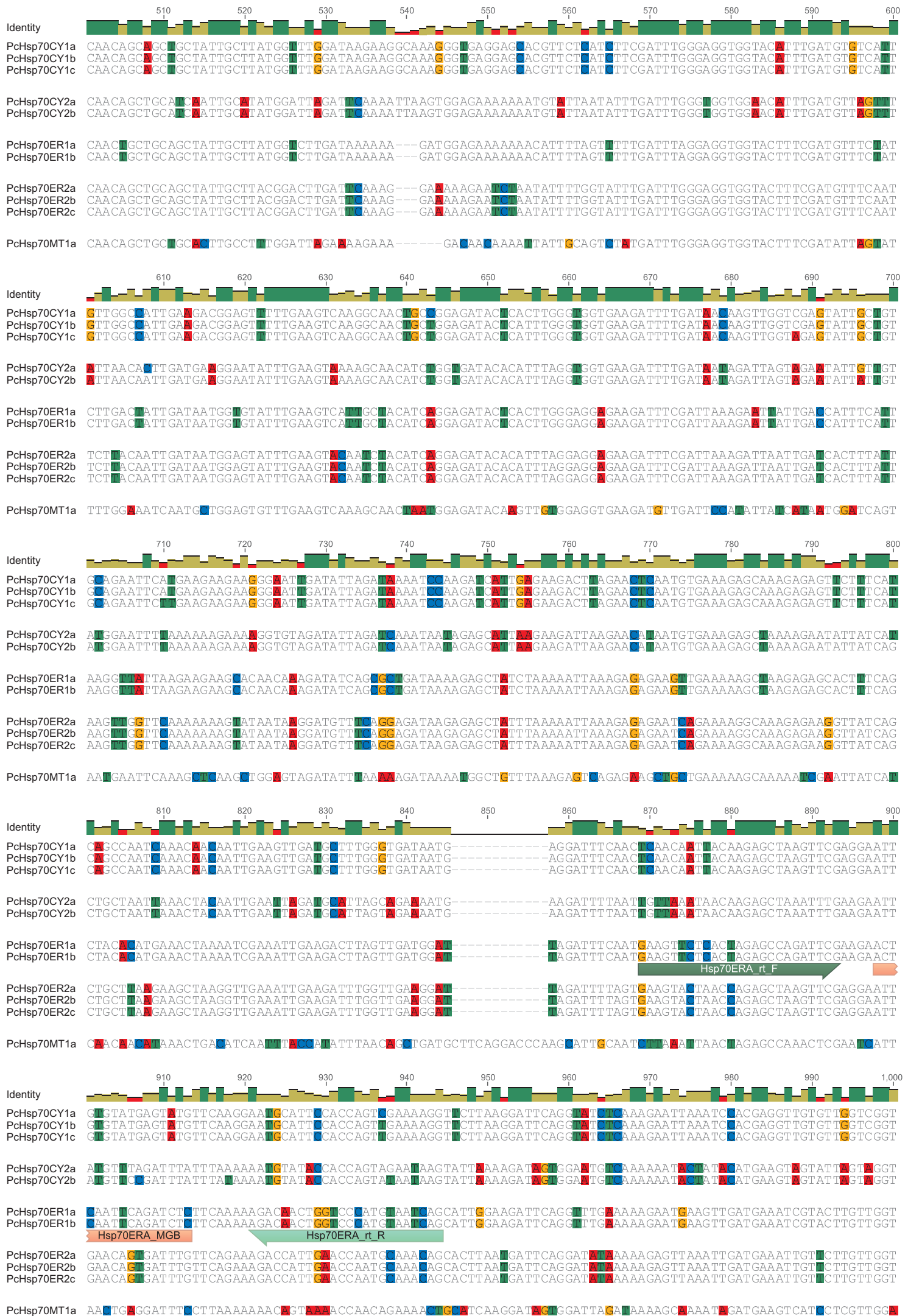

Supplement: Additional file 6: Figure S3 — Paramecium caudatum Hsp70 nucleotide sequence alignment with indicated binding sites for primer and MGB™ TaqMan® hydrolysis probes. [file 1471-2148-13-49-S6.pdf]
